# Interstate Reciprocity in Medical Licensure

**Published:** 1906-12

**Authors:** Samuel G. Dixon

**Affiliations:** Commissioner of Health; Pennsylvania Department of Health, Harrisburg


### Interstate Reciprocity in Medical Licensure.

---

**Health Commissioner Dixon, of Pennsylvania, points out what he considers an insuparable obstacle to reciprocity. The New York Medical Statute removes this barrier.**

Editor Buffalo Medical Journal.

Sir: Your issue for the present month contains an interesting discussion of the question of Interstate Reciprocity in Medical Licensure by yourself and Dr. Howard J. Rogers, First Assistant Commissioner of Education of the State of New York.

Allow me to present what appears to me an insuperable obstacle to such reciprocity, not so much from an academical or educational as a moral standpoint. This consists in the fact that at the present time a medical license is in Pennsylvania, at least, irrevocable.

In this State a man may receive his license and subsequently be convicted of criminal malpractice, serve his time in prison, and then be turned loose upon the community to flaunt his license in the face of an unsuspecting public.

Under these circumstances the New York Regents would scarcely feel justified in accepting a license issued by Pennsylvania without rigid scrutiny of the entire career of the applicant.

There should be some authority designated by law in whom should be confided the power of revoking a license where a licentiate has been convicted of crime. I would suggest tentatively that the Supreme Court of a state would be an appropriate body to be charged with such power. When a license has been revoked, notice of the fact should be at once communicated to each of the State Licensing Boards in reciprocity with the state revoking the same, accompanied with a statement of the reasons for such action.

SAMUEL G. DIXON,
*Commissioner of Health.*

PENNSYLVANIA DEPARTMENT OF HEALTH,
HARRISBURG, OCTOBER 20, 1906.

---

NOTE BY THE EDITOR.—Dr. Dixon's letter is published as an item of interest, but the fervor of his contention is weakened so far as this state is concerned by the fact that Sec. 10 of the New York Medical Statue gives the regents the power to revoke a license for cause, upon the recommendation of the state board of Medical Examiners. This power has been exercised on several occasions, and applies to the endorsement of the license of another as well as to granting an original license to practice.

